# Men’s experiences of suicide bereavement: a qualitative study of psychosocial impacts and coping

**DOI:** 10.3389/fpubh.2025.1613951

**Published:** 2025-05-30

**Authors:** Karl Andriessen, Nina Logan, Shelley-Anne Ball, Tim De Goey, Dianne Currier, Karolina Krysinska

**Affiliations:** ^1^Centre for Mental Health and Community Wellbeing, Melbourne School of Population and Global Health, The University of Melbourne, Melbourne, VIC, Australia; ^2^StandBy Support After Suicide, Maroochydore, QLD, Australia

**Keywords:** bereavement, grief, males, men, mental health, postvention, suicide, trauma

## Abstract

**Introduction:**

Men bereaved by suicide have an increased risk of adverse psychosocial outcomes, including mental health problems and suicidal behaviour. Despite the potentially strong impact of suicide on their life, little is known of how men experience and cope with grief after suicide. The study aimed to investigate men’s experiences of the psychosocial impacts of and coping with suicide bereavement.

**Methods:**

Adhering to the Consolidated Criteria for Reporting Qualitative Research, we designed a study involving semi-structured interviews with a purposive sample (*N* = 34, *M*age = 49.44 years) from across Australia. The transcripts of the interviews were subjected to a codebook thematic analysis.

**Findings:**

The analysis identified three themes: (1) immediate reactions, (2) psychosocial impacts, and (3) psychosocial coping. The findings are underscored by the profound, multifaceted impacts of suicide bereavement, from immediate emotional reactions to long-term mental health effects and trauma. Suicide bereavement frequently disrupted participants’ close relationships and their role as a carer. Participants’ diverse coping strategies included maintaining a bond with the deceased, seeking distraction, or channelling grief into action-oriented approaches. While some potentially maladaptive strategies, such as overworking or substance use, provided temporary relief, other strategies led to personal growth, with some men using their experience to support others or raise awareness about suicide prevention and postvention.

**Conclusion:**

The findings indicate that support must focus specifically on men’s experiences of grief after suicide, and appeal to their coping strategies and feelings of responsibility and role as a carer. Further research is urgently needed to establish best practice to support this population, vulnerable to mental health problems and suicidal behaviour.

## 1 Introduction

Suicide bereavement can present profound and unique challenges for men, including heightened risk across mental, physical, and social domains. Men bereaved by suicide experience a significantly higher risk of suicide compared to non-bereaved men, with some evidence suggesting this risk exceeds that experienced by individuals bereaved by other causes (1, 2). Men bereaved by a partner’s suicide are at increased risk of suicide, mental health issues, substance use disorders, and hospitalisation for a range of health issues (3–5). Mental health outcomes such as posttraumatic stress disorder (PTSD), mood and anxiety disorders, and self-harm are considerably more prevalent in men bereaved by suicide than in non-bereaved men. These men are also more likely to seek psychological therapy and be hospitalised for psychiatric conditions (4).

Men bereaved by suicide also navigate a complex emotional landscape. Guilt, self-blame, and isolation are central to their experience, as they often wrestle with a sense of responsibility for the death and endure an intensely personal and frequently solitary grieving process (6, 7). Traditional masculine norms can exacerbate these difficulties, as expectations of stoicism and self-reliance may lead many men to suppress or distance themselves from their grief. This emotional repression can intensify distress and create barriers to accessing critical social and professional support networks (6, 7).

Men’s coping mechanisms may further reflect the interplay between their grief experiences and personal and societal expectations of masculinity. The literature suggests that avoidance-based strategies, such as overworking or engaging in high-risk behaviours, are common and may serve as a means of emotional regulation consistent with ideals of control and resilience (8, 9). While some men eventually seek support through community networks, others may remain isolated, missing opportunities for connection and shared understanding with those who have had similar experiences (8, 9).

The impacts of suicide bereavement can also extend to men’s relationships and physical health. Men bereaved by a partner’s suicide may experience increased rates of disability, somatic health issues, and difficulties in maintaining family relationships (4). Some adopt the role of protector within their families as a coping strategy, which can offer a sense of purpose but also contribute to distress by diverting attention from their own grief and emotional needs (8, 9).

Despite these far-reaching effects, men bereaved by suicide are less likely to access formal support services or resources than women in similar circumstances, highlighting a significant unmet need (10). Research focusing specifically on suicide bereavement in men is urgently required to better understand their experiences of suicide bereavement, coping mechanisms and help-seeking behaviours, which may guide the development of tailored support services. This study aimed to address this gap by investigating the experienced psychosocial impact of and coping with suicide bereavement in men. In this study, grief refers to the psychosocial impact of bereavement, and coping refers to the strategies used to manage grief (11). Findings regarding experiences with help-seeking will be reported elsewhere.

## 2 Methods

### 2.1 Study design and sampling

We conducted the study according to the Consolidated Criteria for Reporting Qualitative Research (12). We recruited a purposive sample of men bereaved by suicide throughout Australia, between June and July 2024. StandBy Support After Suicide (StandBy), a national suicide postvention service, disseminated the study announcement through their local service delivery sites and collaborating centres. The researchers disseminated the announcement to various (suicide) bereavement services, and professional organisations of clinicians and counsellors. The announcement was also shared with non-government organisations and community-based men‘s organisations, and through social media to reach non-help-seeking men. Snowball recruitment was used by inviting participants to share the announcement with other potential participants. All potential participants were required to contact the researchers. The sample was stratified to include help-seeking and non-help-seeking participants from various locations. Participants were offered a $30.00 AUD gift voucher as reimbursement.

Participants could choose between taking part in a semi-structured individual telephone/online interview, or an online or in-person group interview. Group interviews would be organised if more than one participant expressed interest and would be held at various locations in Australia at the premises of StandBy and co-facilitated by a StandBy lived experience facilitator. Research has shown that individual and group interviews complement each other regarding the breadth and depth of data collected on sensitive topics (13). Offering multiple methods also aimed to allow more potential participants to take part in the research (14), an approach successfully used in previous studies (15, 16). At the end of the interview, participants were invited to take part in an evaluation of their research participation by filling out a short anonymous questionnaire over the subsequent 2 weeks. The findings of this evaluation will be reported elsewhere.

### 2.2 Inclusion criteria

Eligible participants had to: (i) identify as man/male, (ii) be aged 18 years and over, and (iii) be bereaved by the suicide of a close person (such as a family member or friend) at least 6 months before participation. The ‘six months’ criterium was included to avoid recruiting participants who might still be in crisis after the bereavement, and we have used it successfully in previous qualitative suicide bereavement studies [e.g., (15, 16)].

### 2.3 Sample

Based on the literature on information power (17), and our experience with qualitative suicide bereavement research [e.g., (15, 16)], we estimated the required sample size at approximately 15 participants. Nonetheless, given the shortage of research with this population, it was *a priori* decided to provide an opportunity to all eligible participants to participate within the available time window (June–July 2024). As the analysis of the final interviews did not yield new codes, we determined that the sample size was adequate.

A total of 48 potential participants contacted us and 34 from across Australia participated in the study. Reasons for not participating were being unavailable (*n* = 7), being ineligible (i.e., being bereaved less than 6 months: *n* = 3; not identifying as man/male: *n* = 1), and participant withdrawal (*n* = 3).

The mean age of participants (*N* = 34) was *M* = 49.44 (*SD* = 13.76, range 23–71 years). The average time since the bereavement was *M* = 8.62 (*SD* = 10.12, range 0.5–43 years). There was no significant correlation between age of participants and time since bereavement.

About one in three participants (38.2%) had lost a child to suicide (son *n* = 9, daughter *n* = 4), one in six (17.6%) had lost a sibling (brother *n* = 5, sister *n* = 1), and one in six (17.6%) had lost a parent (father *n* = 4, mother *n* = 2). Four participants had lost a spouse or partner (11.8%), and five participants (14.7%) had lost another family member (*n* = 1) or friend (*n* = 4).

If participants had lost more than one person to suicide, the bereavement they felt most important was recorded. Six participants spoke about more than one person who had died by suicide. These included the participants’ mother, mother-in-law, grandfather, friends, and colleagues. A total of 26 participants (76.5%) had used formal support (i.e., from services or counsellors) after the bereavement. The others had contacted a service only once or had not sought formal support.

### 2.4 Data collection

We developed a semi-structured interview guide, adaptable for individual and group interviews, with open-ended questions allowing for probing and follow-up questions (18). The lead questions inquired about what helped or hindered coping with suicide bereavement, the needs for support and how to meet such needs, experiences and help-seeking, and what participants perceived as effective in help-seeking. The interview guide was based on a strengths-based approach to validate the lived experience and agency of the participants, to explore protective factors and what is helpful and supportive from their perspective in terms of coping and help-seeking after the bereavement.

Due to limited participant interest in group interviews, only individual interviews were conducted. Researcher K.A. conducted all interviews and recorded field notes after the interviews. Interviews lasted on average *M* = 50.69 min (*SD* = 10.53, range: 27–68.5). Nineteen interviews were conducted via Zoom (55.9%), and 15 via telephone (44.1%). The mean duration of telephone interviews was shorter than Zoom interviews (44.17 min vs. 55.84 min, *p* < 0.001). Telephone interviews were often scheduled during the lunch break when participants were at work, which may, at least partly, explain the difference in duration. The interview duration was not associated with age of participants or time since bereavement. All interviews were audio-recorded and professionally transcribed with transcriptions checked for accuracy.

### 2.5 Research team and reflexivity

The study was based at the Centre for Mental Health and Community Wellbeing at The University of Melbourne, a national and international leader in suicide and mental health research. The lead researcher, K.A., is a social worker with ample experience in qualitative (suicide) bereavement research. N.L. is an early career researcher focusing on gender and mental health promotion. S.B. is a clinical psychologist and Clinical Director at StandBy Support After Suicide. T.D.G. is a Partnership Coordinator and lead for Men Priority Population Group at StandBy Support After Suicide. D.C. is an Associate Professor specialised in evaluating suicide prevention service delivery, and men’s mental health research. K.K. is an experienced suicide-related research psychologist and psychotherapist. Several team members have lived experience of suicide. The research team met regularly to ensure consistency throughout the study.

### 2.6 Data analysis

We uploaded the deidentified interview transcripts in NVivo14 (19) and conducted a thematic analysis (20). Two researchers (N.L. and K.A.) created a codebook based on independent analysis of three transcripts (21). Next, one researcher (N.L.) coded the remaining transcripts while holding regular discussions with the lead researcher. The same two researchers further analysed the data through an iterative process based on the six steps described by Braun and Clarke (22), adopting an inductive approach to capture the meaning of the data rather than its explicit content. Mind maps (i.e., diagrams) were used as a visual aid to conceptualise potential themes, which were checked against the data before deciding the themes.

## 3 Findings

The findings describe the impacts and experiences of suicide bereavement, including immediate reactions (theme 1), psychosocial impacts (theme 2), and psychosocial coping (theme 3). Table 1 summarises the themes and subthemes.

**Table 1:** Summary of themes and subthemes.

Theme	Subtheme	Example quote
Theme 1: Immediate reactions		“Everything I thought I knew or everything I thought about my future and what that looked like, that just completely shattered and came apart. My understanding of who I was and my place in the world and all of that just completely fell apart.” (P65).
Theme 2: Psychosocial impacts	1: Grief	“I was zeroed in white hot rage with a sniper rifle accuracy of exactly who I blamed for the action [son’s name] took on that Monday morning.” (P49).
	2: Mental health, trauma and suicidality	“Six weeks later, I ended up in the [name] Hospital, the psychiatric ward, because I did not want to be here, and I felt really strange, so I knew that that was the safest place to go.” (P40).
	3: Relationships with family and romantic partners	“… parents who lose a child, 80% of us get divorced … Marriages fail three or four years down the track, including mine … So, it’s like great pain on top of pain that we have got to deal with.” (P02).
	4: Social isolation	“… It was something that was in the air, this feeling that everyone knew how dad passed, but it was never really openly talked about. For me, having someone to be able to start that conversation without the burden having to be on me to actually bring it up would have been nice.” (P51)
Theme 3: Psychosocial coping	1: Continuing bonds and connection	“I was quite enjoying coming here because this is where his ashes were … I said to [my partner] I need to make a road trip. I drove back up here again.” (P11).
	2: Reflection and restoration	“I’ve discovered art since and that helps me a huge amount … you get out there and it’s like, bang, yeah, okay, I feel better.” (P02).
	3: Avoidance and distraction	“…you are just going about your day and not really taking in everything or feeling a lot … It’s just feeling blank, feeling not very present, putting on a façade, like putting on a bit of a mask and being like, yeah, it’s all good. But then, deep down…” (P51).
	4: Active and practical	“I feel like I’m making a difference … I’ve got to focus that anger and energy into a direction, otherwise it’s probably going to consume me.” (P02).

### 3.1 Theme 1: Immediate reactions

Participants described the profound and wide-reaching immediate effects of the bereavement, impacting every aspect of their lives. They reported an intense sense of disorientation and identity loss, as their understanding of themselves and their future was fundamentally altered. One participant shared: *“Everything I thought I knew or everything I thought about my future and what that looked like, that just completely shattered …*. *My understanding of who I was and my place in the world and all of that just completely fell apart.” (P65)*. In some cases, this severity of impact was linked to the unexpected nature of the death by suicide; *“the immediate feeling that I had was shock. It felt like I did not actually get to go through the proper grieving process for quite a long time. I was in that shock for a long time.” (P51)*.

However, when the suicide was less unexpected, the impacts could still be substantial: *“I knew it was going to happen. That does not make the loss any easier and it does not make it any less shocking than when it happened.” (P15)*.

A few participants described an immediate acceptance, particularly where they perceived the person who died by suicide had been suffering prior to their suicide; *“I do not think I was ever really … that sad because … he found life agonising I think and so very quickly I realised that … he made a decision that gave him peace. I saw him the day before and he was the calmest and happiest I’d seen him in over a decade.” (P20)*.

### 3.2 Theme 2: Psychosocial impacts

This theme describes the profound personal effects of suicide bereavement on participants by exploring the emotional, psychological, and relational challenges they experienced. The impact of grief, mental health struggles and trauma, as well as the impacts on relationships are central to understanding how men navigate life after losing someone to suicide.

#### 3.2.1 Subtheme 1: Grief

For many participants, emotional experiences of grief were dominated by feelings of anger, guilt and confusion at the intentionality of the death by suicide. This anger could be directed at the person who died by suicide, other people who they felt were responsible for their death, or systems that failed them; *“I was zeroed in white hot rage with a sniper rifle accuracy of exactly who I blamed for the action [son’s name] took on that … morning.” (P49)*. Reflections on feelings of guilt frequently stemmed from participants attributing some degree of responsibility for the suicide to their own actions, such as ending a romantic relationship with their partner shortly before their death. Others experienced guilt relating to their own perceived inaction; they thought they had not done enough to provide support or prevent the suicide: *“I felt like a fair bit of guilt about the fact that I had not realised he was struggling so much, felt like I should have been able to help him, and I should have known … if I had known, then he would not have done it.” (P03)*.

Many participants described oscillating through different strong emotions, particularly immediately following their bereavement by suicide, that felt completely outside of their control: *“… you could’ve come and knocked on my door and there would’ve been one of four different [versions of myself] there at the door … I could be in a good mood, I could be okay with life, but I do not want to talk about [son’s name]. I could be okay, but I need to talk about [son’s name]. Or I could be not in a good place, and I still need to talk about [son’s name], or I’m in a really bad place and I cannot go there at all. It’s not like I could ever pick that’s who I was that day. It’s just that’s who I happened to be that day.”* (P66).

#### 3.2.2 Subtheme 2: Mental health, trauma and suicidality

For many men, suicide bereavement precipitated significant declines in their mental health and wellbeing. In some cases, the impacts were immediate and severe, as illustrated by a participant who entered voluntary psychiatric hospitalisation shortly before his partner’s memorial: *“… six weeks later, I ended up in the [Hospital], the psychiatric ward, because I did not want to be here, and I felt really strange, so I knew that that was the safest place to go.” (P40)*. Others experienced mental health problems for the first time, including diagnoses of depression, anxiety, PTSD, and prolonged grief disorder (complicated grief): *“I was feeling just completely overwhelmed. I had never experienced mental ill health before in terms of depression and anxiety, and I learned ways to cope with those.” (P12)*.

Experiences of PTSD were common among participants, particularly those who found the body of the deceased. Trauma could be triggered in many ways, even by searches for suicide bereavement support services: *“… it’s quite traumatic. So, even to look up suicide bereavement services, or read any research on it and stuff like that, there’s still that slight trigger there.” (P51).* While some men experienced recovery from these mental health problems, they often felt that *“the wounds will always be there, and they always have ripple effects” (P18)*.

While participants were not directly asked to disclose their own suicidality, eight participants mentioned experiences of suicide ideation following their bereavement by suicide. Half of these individuals knew more than one person who had died by suicide, and all but one had lost a first-degree relative to suicide. A man whose child had died by suicide related that experiencing suicidality following bereavement was common among others in his support group for suicide-bereaved parents: *“… the amount of times I’d speak to other bereaved parents and they would say that they are suicidal after the death of a child. For me, it’s a normal response. It’s such an impossible loss; to be thinking about the same thing, it just seems like a natural response … the amount of parents who attempt or complete suicide after the death of a child … I would suspect it would be very high …” (P02).*

Some men experienced passive suicide ideation, sometimes linked to changed ways of thinking about mortality following their experience of suicide bereavement: *“I think that that sense of fear of death … has abated but I still spend plenty of time thinking when will it be my turn to die, when will I get that opportunity to die?” (P17)*. Others attempted suicide, in some cases repeatedly: *“Tried to take my own life … [I was] in a black hole, yeah, really deep. Took me a long time to get out of it.” (P43)*.

The degree to which participants attributed experiences of mental health problems and suicidality to their experience of suicide bereavement varied and was often mediated by their relationship to the deceased and other impactful or traumatic events in their lives: *“It’s all bundled up to be honest. I think it’s all my traumas from throughout my life. So yes, definitely [my brother dying by suicide] plays a part in it but it’s also my brain surgeries and my disability and stuff like that. It all plays a part” (P20)*.

#### 3.2.3 Subtheme 3: Relationships with family and romantic partners

Many participants experienced a substantial impact on their relationships. For those whose bereavement by suicide occurred further in the past, there was often a reflection that the mental health and trauma-related effects of the bereavement were inseparable from its impacts on their relationships with those close to them, with both aspects compounding their distress. *“I think where I’m not recovered is the impact on my whole family … You can work on stuff yourself, but when it’s a relationship that’s harmed and there’s multiple different actors and dimensions to it, I think that’s the ongoing effect from a traumatic event … that one person’s journey [will lead them to] look at [the experience of suicide bereavement] in one particular way [that] is different to somebody else’s. You wind up with quite different world views about things.” (P26).*

Many fathers bereaved by the suicide of a child reported the breakdown of their relationship with their romantic partner (mainly the mother of the deceased child). They described this breakdown as compounding the grief from their child’s death: “*… parents who lose a child, 80 % of us get divorced … Marriages fail three or four years down the track, including mine … it’s like great pain on top of pain that we have got to deal with.” (P02). Participants* attributed these relational difficulties to various factors, including their own grief response: *“[My child’s suicide] had a really big impact on my marriage … I guess I was probably a pretty challenging person to be around going through that grief and bereavement.” (P65),* use of alcohol to cope: *“… my wife and my other kids, they were going to leave me. They said if I do not stop drinking and that, they are going to go.” (P43)*, or insufficient provision of social support: *“I did not talk it through much with my partner, and probably was a factor in a year or so later us separating.” (P54)*.

Participants bereaved by suicide as children often experienced a profound loss of emotional support, particularly when the deceased parent had been the primary caregiver, and the surviving parent was unable to meet their needs. *“… my dad was the type of guy that did not really talk about his emotions much and did not really open up. I wasn’t able to talk with him about it … I’m an only child. My mum was the parent that did most of the stuff for me, most of the taking me to school, sporting events, music lessons, whatever … To lose that mother, caregiver role, all I had was this pragmatic dad that I did not really need” (P65)*.

Regardless of the parenting role of the person who died by suicide, men bereaved as children often reflected on how their surviving adult family members were less able to provide care and support due to the impacts of their own grief: *“my mum was very badly affected by my dad’s death … she really wasn’t able to really be a supportive parent herself. So it’s like both parent figures just were gone in a day, that whole structure changed. I was on my own and had to come to grips with that. So I think that compounded things.” (P26). Some participants felt that the absence of formal and emotional support for both themselves and their grieving family members highlighted a missed opportunity for intervention: “I could have probably done with a bit of counselling and a bit of guidance. I think the people around me in my family could have done with a lot of support, and that would have supported me as a child, but it did not happen.” (P02).*

#### 3.2.4 Subtheme 4: Social isolation

Many participants reported often feeling socially isolated. This was more common, or intensified, for men who typically did not share personal feelings or issues with those close to them: *“I do not have any close friends where I feel comfortable [talking about my bereavement] … We focus on things that are good. We do not talk about how stressful and hard work was or our relationship problems … We deal with our stuff outside of the social circle …*” *(P13).* This self-isolation was often justified by the conviction that they were alone in their experiences and that others could not understand their emotions or thoughts: *“I’ve got to figure out my own solution … I know lots of people experience it, but at the time, it just felt like no one’s going to understand …” (P51).*

Some men who did seek social support found it to be limited, particularly when trying to connect with family and friends who were also grieving. Some described how collective grief made meaningful conversations difficult: *“[My wife] and I spoke all the time but we were too immersed in our own grief and the three remaining siblings and the two daughters-in-law, they were all just coping and dealing [by] themselves. … It was trying to take apart the details leading up to the event and trying to understand the why collectively. But I did not get a sense that they were ever going to give me what I needed …” (P49).* Over time, this participant withdrew from these conversations, fearing he would become a burden.

Beyond family, men also described a sense of social isolation in the broader community, where their grief felt known but unspoken. The perceived stigma surrounding suicide contributed to this silence: *“… the stigma around suicide, it just made it really difficult to actually have those open conversations. It was something that was in the air, this feeling that everyone knew how dad passed, but it was never really openly talked about. For me, having someone to be able to start that conversation without the burden having to be on me to actually bring it up would have been nice.” (P51)* Some who attempted to initiate discussions found that others withdrew, particularly following the immediate aftermath of the death: *“a lot of people do not necessarily want to talk much about it. Once there’s the funeral …, it just quickly goes away. You can see some people shy away if you try to engage. Which is what I found. I did not think my friends would do that. But they did not want to engage much.” (P11)*.

For some, this lack of engagement led to the breakdown of friendships, deepening their isolation. In response, some sought out support groups to find a sense of connection with others with similar experiences: *“a lot of people I have not seen for years … We did not have a lot of friends after [our son died by suicide]. I do not know if it was shame or they did not want to talk about it or whatever, but yeah, we sort of become a bit isolated … It’s hard because there’s other people that are in that situation, that you could support each other, so that’s why I started up the support group … so that we could all support each other.” (P43).*

### 3.3 Theme 3: Psychosocial coping

This theme illustrates participants’ diverse coping mechanisms following suicide bereavement, informed by both their personal preferences and complexity of their emotions. Some strategies aimed to maintain an ongoing connection with the deceased, while others focused on reflection, self-care, or active efforts to address their grief. Participants also addressed engaging in escapism and distraction, including substance use, overwork, and emotional detachment, as temporary means to manage overwhelming feelings. Additionally, practical and problem-focused coping often provided a sense of agency and control amidst the chaos of grief.

#### 3.3.1 Subtheme 1: Continuing bonds and connection

Many participants engaged in acts of memorialisation to honour and maintain a connection with the deceased. This often involved visiting the place of interment, with one participant describing the comfort they found in returning to this location: *“I was quite enjoying coming here because this is where his ashes were … I said to [my partner] I need to make a road trip. I drove back up here again.” (P11)*. Others found solace in talking about the deceased with friends and loved ones, acknowledging their positive qualities and shared memories: *“It’s also about acknowledging the good parts of the mates that are lost … being able to talk about them and with your loved ones, and being able to talk with your friends about that.” (P46)*. In some cases, participants continued their bond with the deceased by engaging in conversations with them, using these interactions to preserve a sense of connection*: “I have quite deliberate structured conversations with my dad … about things that I think I would have conversations with him about if he was alive.” (P26)*.

Many participants expressed that publicly acknowledging their loved one’s mental health problems and death by suicide was integral to honouring their memory. For them, speaking openly was not only a means of processing their own grief but also a way of remaining true to the person they had lost: “*we were not going to hide that [brother’s name] had a mental illness. We were not going to hide that this is the decision that he made … If we had done that, I feel like that that would have been a disservice to his memory. It would have been dishonest towards [him] and about the person that he was.” (P16)*.

This openness often led to unexpected connections with others, fostering reciprocal sharing of grief and healing: *“if in a conversation, an opportunity comes up to just mention the fact that I’ve lost my daughter, it’s amazing how the conversation opens up and the number of people who then reveal their own experiences, whether it’s a friend of theirs who took their life or their own struggles with mental health or a family member … that’s been also quite a cathartic experience, feeling that through our tragedy we have been able to help other people process or grapple with their own difficulties.” (P42).*

#### 3.3.2 Subtheme 2: Reflection and restoration

Many participants described the positive impacts of engaging in self-care activities to help cope with their grief. The specific nature of these activities varied based on personal interests, but the benefits were often linked to staying busy, connecting with others, and finding mental relief. One participant highlighted the immediate sense of calm that came from their creative pursuits: *“I’ve discovered art since and that helps me a huge amount … you get out there and it’s like, bang, yeah, okay, I feel better.” (P02)*. Others engaged in self-care by taking time away from work or study.

In addition to leisure activities, self-compassion and dedicating time to care for themselves emotionally were crucial in managing the complex grief of suicide bereavement. This involved acknowledging difficult emotions and allowing space to process them: *“The most helpful thing in my grief was recognising the days that I was having most trouble and just taking time for myself, being gentle with myself and honouring my emotions” (P16)*. Over time, this internal journey led some men to reach a sense of acceptance, as they gradually allowed themselves to experience happiness again: *“I’ve spent a lot of time feeling like I wasn’t entitled to feel any kind of joy … so it’s a matter of gradually learning to give yourself permission” (P17).*

Participants described how self-care and self-compassion were coping strategies that often led to greater emotional regulation and improved relationships with others: *“… if I feel sad or angry now, I just try to manage it and I do not let that linger for a day. I try not to let that affect my relationship with my partner … Managing those emotions and feelings rather than feeling down for an extended period of time or pushing people away when I do not feel like talking or communicating to them. Just a reminder to yourself that not everyone will understand what you are going through so you cannot blame them.” (P69).*

#### 3.3.3 Subtheme 3: Avoidance and distraction

Participants reflected on how some of their coping strategies were potentially maladaptive. Many described experiencing a sense of “numbness” for weeks or months following the bereavement. As this numbness began to fade, it was often replaced by a sense of emotional detachment: *“… you are just going about your day and not really taking in everything or feeling a lot … It’s just feeling blank, feeling not very present, putting on a façade, like putting on a bit of a mask and being like, yeah, it’s all good. But then, deep down … more of that sadness came through.” (P51)*.

For some, this emotional restraint felt socially enforced, particularly by other men around them. One participant shared how masculine social norms restricted emotional expression, even among close friends: *“It was a month [after] my wife passed away … We’re drinking tequila at the kitchen table … The biggest thing in my life was the loss of my wife. Do you know what subject we spent half an hour talking about? Whether golf was a game or a sport. I look back on it now and feel that moment was a betrayal … I was screaming inside to deal with issues.” (P47).*

Substance use was also a common strategy: *“I drank a lot for probably two or three months after, every night I drank a lot of alcohol.” (P16)*. While offering temporary relief, participants felt that the use of alcohol or drugs to cope could also negatively impact their lives: *“I drank a lot. I smoked a lot of pot … overdid it a few times and just realised that it was really setting me on a bad track …” (P26),* or strain family relationships: *“My wife was sort of looking after [our surviving children] … whereas I was on the piss and taking drugs, that was my outlet.” (P43)*.

Overwork was frequently mentioned as a distraction from the emotional pain of suicide bereavement, often viewed similarly to substance use: *“I think I coped with it by just working like a lunatic and drinking way too much alcohol.” (P02)*. Overworking could also provide a sense of control or escape or be a way to honour the memory of the deceased. For example, men who pursued mental health advocacy or awareness felt they were continuing the legacy of their loved one: *“… my partner at the time said why are you doing that, why are you taking on more, you have just lost your daughter. Why are you taking on more and doing these things? I tried to explain that it was partly a reaction to losing my daughter, that she was someone who stepped up and did stuff, and I sort of felt like I needed to do that.” (P54).*

#### 3.3.4 Subtheme 4: Active and practical

Several participants adopted a problem-focused approach to coping with their grief, particularly those who identified with traits of *“self-reliance and stoicism … I’ve got to figure out my own solution to the problems, and no one else was really going to be able to understand or help” (P51)*. These participants often directed active coping efforts towards the source of their grief - suicide - with many engaging in suicide prevention efforts, including fundraising, or awareness raising activities: *“I feel like I’m making a difference … I’ve got to focus that anger and energy into a direction, otherwise it’s probably going to consume me.” (P02).*

Some participants used their own lived experience of suicide bereavement to support others: *“I am a lived experience advocate … I do men’s mental health training and workshops.” (P47)*. Others entered mental health-related fields, driven by their personal loss: *“I have learned so much since my dad passed away, about myself … I have a purpose of helping other people with their mental health in general, but specifically people who have been bereaved by suicide …” (P64)*.

While these active coping strategies often helped men feel “functional,” some participants acknowledged that deeper emotions remained unaddressed, particularly when these strategies were used in lieu of accessing formal support services. As one participant reflected: *“I had basically coped for 10 years being crazy, but functional” (P47)*. Another further identified this need for agency as a distinctly masculine coping strategy, shaped by societal expectations of men’s roles: *“It’s like I got control. I can fight. I can yell … Men, in terms of control, influence, and power, have traditionally been afforded more control over outcomes in* var*ious areas of society … I think that does play into how we approach agency in grief and bereavement” (P65).*

## 4 Discussion

This study aimed to investigate how men have experienced the impact of the suicide of a close person, and their grief and psychosocial coping after the bereavement. The study fills a major gap in informing best practice in supporting this population. Thematic analysis identified themes in three key domains: bereaved men’s immediate reactions to the death by suicide; the impacts including grief experiences, their own mental health and suicidality, shifts in relationships with others; and the way they coped with suicide bereavement.

### 4.1 Immediate reactions and impacts of suicide

All participants in this study talked about how experiencing bereavement by suicide was a life-changing event with immediate and long-term impacts. Initial reactions included feelings of shock, disbelief and sadness, which are commonly reported in the suicide bereavement literature (23, 24). For many of the participants, suicide bereavement was a traumatic experience, shattering their dreams for their future. Consistent with the general suicide bereavement literature, many participants also reported feeling angry towards themself or the deceased person; they struggled with trying to understand why the suicide had happened, and feelings of guilt for not being able to foresee and prevent the suicide (23, 24). Struggles with meaning-making have also been reported in studies with men bereaved by other traumatic causes [e.g., (25)].

Many study participants had experienced the onset or exacerbation of mental health problems after the bereavement, such as depression, anxiety and PTSD. For a few, this resulted in psychiatric hospitalisation. The literature indicates that suicide bereavement is a risk factor for mental health problems, especially when there are pre-existing mental health or relationship problems (1, 26, 27), as was the case for some of our participants. Suicide-bereaved men compared to non-bereaved men have been found to have an 80% higher risk of mental health problems over 5 years after bereavement, and a double risk for psychiatric hospitalisation (4). Studies that compared suicide-bereaved men and women tend not to find significant differences in mental health problems; however, it has been noted that increases in scores of mental health problems in suicide-bereaved men could be masked due to higher baseline scores in women (11, 28).

Experiencing suicidal ideation or suicide attempts was a concern for several participants. Suicide bereavement, especially after suicide of a close family member, is an important risk factor for suicidal ideation, attempted suicide and death by suicide for men and women (27, 29, 30). However, there is evidence from the literature (1), including large population studies (31), that men bereaved by suicide are at increased risk of hospitalisation for suicide attempts and suicide mortality compared to non-bereaved men and those bereaved by other causes (1, 2). As attempted suicide is a major risk factor for suicide, suicide prevention efforts should be targeted at this high-risk group of suicide-bereaved men.

Participants reported a strong impact of suicide bereavement on their relationships and roles as a family carer, such as a father or spouse/partner. Some participants also felt a lasting impact of losing a parent by suicide during their childhood. While the importance of relationships was already noted in pioneering suicide bereavement literature (32), few studies have examined this issue (33). A recent study with suicide-bereaved adolescents and parents reported how bereavement disrupted the family equilibrium creating a limbo maintained by individual differences in grieving needs and grief reactions (34). It has also been noted that externalising problems in bereaved children are more associated with bereaved mothers’ distress and their parenting and communication, than with those of fathers, highlighting their potential importance as a family carer (35).

Loneliness is associated with suicide attempts after sudden bereavement, including suicide bereavement (36). In suicide-bereaved men, feeling disconnected from others can compound the experiences of grief after suicide (7). This was described by many study participants who experienced relationship breakdowns and feelings of loneliness after the bereavement. While some felt disconnected from their social networks, for others it appeared to be a choice to self-isolate or be selective in sharing their grief. This finding expands our understanding of this aspect of suicide bereavement by describing how men may deliberately suppress their emotions to protect their families from the weight of their mourning (6). Suicide-bereaved men have attributed their emotional repression to masculine ideals of stoicism and the importance of self-control and being strong to support their loved ones (9).

In addition, men bereaved by a partner’s suicide (4) or a child’s suicide (37) have been found to have higher rates of unemployment and sick leave (4), or absences from work (37) than non-bereaved men, which may decrease opportunities for social interactions and support. As self-disclosure and social interaction may contribute to posttraumatic growth in people bereaved by suicide, interventions may focus on promoting social support (38), though studies specifically with men are needed.

### 4.2 Psychosocial coping

Expression of and coping with grief is deeply influenced by gender and sociocultural expectations. Doka and Martin (39) characterised masculine grief as instrumental or problem-solving, and feminine grief as intuitive or emotional, with one coping style being not necessarily less or more effective than the other. Although gender has a strong influence, it does not determine grief styles. Therefore, they proposed that grief styles represent a continuum between instrumental (action-oriented) and intuitive (affective-oriented) grieving (39). In Western societies it is more commonly expected and accepted that women express and share their grief, and for men to control and conceal it, as confirmed by some of our participants. The suicide bereavement literature indicates that men tend to grieve in secret, bottle up and mask their feelings, and feel uncomfortable publicly expressing their grief feelings (28, 40). Keeping strong to support a spouse or other bereaved family members may lead to delayed grief expression. This may lead to suicide-bereaved men feeling ignored and unacknowledged as a bereaved person, which has also been reported in studies with bereaved fathers (41), and adolescents bereaved by suicide (34, 42).

Participants in our study have used a range of coping strategies including strategies to engage with and/or to avoid their grief. Consistent with the grief dynamics described in the Dual Process Model of coping with bereavement (43), participants adapted and oscillated between loss-oriented and restoration-oriented stressors. Many participants shared experiences of how they actively maintained a bond with the deceased person, for example, by visiting the interment place. This is corroborated by the literature which found that maintaining a continuing bond is mainly a positive experience that may contribute to meaning-making (44). Men may express the continuing bond more privately than women, for example through conversing with the deceased (44).

While some participants took time for self-care and to acknowledge difficult emotions, many participants reflected on how they avoided engaging with their grief, for example by detaching emotionally, finding distractions, or engaging in substance use or work. Other studies have also noted engaging in substance use and work as a coping strategy in men bereaved by suicide (7, 8). Suicide-bereaved men often experience the same range and depth of emotions as women but struggle to identify and express them (9), or they express them differently (28, 40). As stated by our participants and the literature, engaging in work may contribute to regaining a sense of control and meaning, though some men lose motivation for work (45, 46).

Many participants adopted an active and problem-solving coping style, for example by engaging in fundraising, suicide prevention or bereavement support activities. The scarce literature on this topic reported that people (men and women) who had been bereaved by suicide for 5 years or more (i.e., long-term bereaved) had lower scores on mental health problems, higher scores on personal growth, and had taken up more caregiving roles at support group meetings (47). Our findings extend the scarce suicide bereavement literature on this topic (48, 49) as our study indicates that engaging in support and community activities may be particularly beneficial for suicide-bereaved men. It can alleviate feelings of loneliness and provide a way of honouring the deceased person and an opportunity for finding purpose, meaning, and personal growth.

Adopting an active coping style can also be understood in the context of agency and self-empowerment, i.e., taking action to create a new future and find meaning (50). According to the general literature, individuals may engage in active coping strategies such as seeking knowledge by reading books or other resources on suicide and bereavement, helping others through altruistic actions like participating in suicide research, or becoming a peer supporter (50–52). These actions align with the active coping styles reported by our participants, enabling them to manage their bereavement while supporting others simultaneously.

Our findings indicate that the motivation to become a peer supporter is strongly associated with one’s own suicide bereavement experience, the support they have received, and a wish to ‘give back’ by providing support and hope to others (47, 53). Our participants also wanted to ‘make a difference’, for example by tackling silence around suicide, creating public awareness, and contributing to preventing the suicide of others. Studies suggest that most peer supporters tend to experience their involvement positively, but they may also encounter important challenges related to being confronted with others’ suicide grief and navigating group or individual support relationships (49). Nonetheless, the involvement of trained peer workers has been identified as contributing to the effectiveness of suicide bereavement support (54).

### 4.3 Implications

The study findings have important implications for developing support for men bereaved by suicide. Support offered to this population must focus specifically on experiences of grief after bereavement by suicide and appeal to men’s coping strategies (55). Although study participants used various coping strategies, the most prevalent were feelings of responsibility and keeping strong to support others, including spouse/partner or children. Several participants were also aware of the importance of self-care. Therefore, the support offered may appeal to their role as a caregiver and accommodate both instrumental and action-oriented coping. Support services might actively engage with the lived experience of suicide-bereaved men to further develop and tailor support offered to this population (52, 55).

Given the scarcity of research in this field, further studies are urgently needed to examine support and services specifically offered to men bereaved by suicide and their potential to alleviate grief and mental health distress and decrease their risk of suicide (56, 57). Evaluation studies may contribute to establishing the evidence-base of various types of support (including peer and professional support), and to ensure that optimal support is offered to this population, vulnerable to mental health problems and suicidal behaviour.

### 4.4 Strengths and limitations

The study successfully used multiple recruitment channels to recruit a large purposive sample of men who had experienced bereavement due to suicide. Participants varied in age, time since bereavement, type of relationship with the deceased, including non-family members, and location across Australia, which resulted in a rich data set. Nonetheless, a few limitations must be acknowledged. Recruitment of voluntary participants through suicide bereavement organisations may have attracted participants with a more active coping style. Those who were more verbally skilled and felt comfortable discussing their experiences may have been more likely to volunteer than others. Thus, the findings may not reflect the views of men who did not participate. The study relied on self-report of experiences up to more than 30 years previously, which is open to recall bias. The study did not collect data on ethnocultural or sexual diversity, or focus on social support or grief trajectories. Additionally, although participants had ample opportunity to talk about their experiences, it is possible that some important issues were not shared.

## 5 Conclusion

The study investigated the psychosocial impact of and coping with suicide bereavement in men. There is strong evidence of a substantial impact of suicide bereavement on men’s feelings of grief, trauma, mental health, suicidality, and relationships, to the extent that it may affect their feelings of responsibility and care for others such as their spouse/romantic partner or children. The participating men used various coping strategies including maintaining a continuing bond with the deceased, self-awareness and self-care, finding adaptive and/or maladaptive distractions through substance use or work. Some also used active approaches such as becoming a peer supporter which contributes to reclaiming a sense of agency and control amidst the chaos of grief. Further research is urgently needed to establish best practice in line with men’s coping strategies to support this population, vulnerable to mental health problems and suicidal behaviour.

## Data Availability

The datasets presented in this article are not readily available because the ethics approval does not allow sharing original data beyond the research team. Requests to access the datasets should be directed to Karl Andriessen, karl.andriessen@unimelb.edu.au.
